# Prevalence and characterization of class I integrons in multidrug-resistant *Escherichia coli* isolates from humans and food-producing animals in Zhejiang Province, China

**DOI:** 10.1186/s12866-025-03794-y

**Published:** 2025-02-15

**Authors:** Han Jiang, Meijuan Ran, Xinyuan Wang, Qi Chen, Jing Wang, Zhi Ruan, Jingwen Wang, Biao Tang, Jiehong Fang

**Affiliations:** 1 Key Laboratory of Specialty Agri-products Quality and Hazard Controlling Technology of Zhejiang Province, College of Life Sciences, China Jiliang University, Hangzhou, 310018 Zhejiang China; 2 Hangzhou Institute for Food and Drug Control, Hangzhou, 310022 Zhejiang China; 3 Zhejiang Gongzheng Testing Center Co., Ltd, Hangzhou, 310000 Zhejiang China; 4 Department of Clinical Laboratory, Sir Run Run Shaw Hospital, Zhejiang University School of Medicine, Hangzhou, 310016 Zhejiang China; 5 Key Laboratory of Precision Medicine in Diagnosis and Monitoring Research of Zhejiang Province, Hangzhou, 310016 Zhejiang China; 6 Key Laboratory of Systems Health Science of Zhejiang Province, School of Life Science, Hangzhou Institute for Advanced Study, University of Chinese Academy of Sciences, Hangzhou, 310024 Zhejiang China

**Keywords:** Class I integrons, Gene cassettes, Multidrug-resistant *Escherichia coli*, Food-producing animals, Human patients

## Abstract

Class I integrons have garnered significant attention due to pivotal roles in the dissemination of antimicrobial resistance genes (ARGs), which impose risks to public health and food safety. Here, the prevalence and characteristics of class I integrons in *Escherichia coli* isolates derived from food-producing animals and human patients were assessed. Of 721 *E. coli isolate*s collected from human patients (113), pigs (298), and poultry (310), 93 (12.90%) carried the class I integrase gene (*intI1*). Multilocus sequence typing identified 39 sequence types from 93 *intI1-*postive isolates, including three novel types. Sequence analysis revealed that 59 classical class I integrons encompassed six distinct gene cassettes arrangements [*dfrA17*-*aadA5*, *dfrA12*-*aadA2*, *dfrA1*-*aadA1*, *dfrA7*, *aac(6’)*-*Ib*, and *aadA1*-*aac(3)*-*VIa*]. Six insertion sequences (IS*1*, IS*6*, IS*21*, IS*91*, IS*110*, and IS*256*) and one transposon (Tn*3*) were harbored in proximity to the integrons. A comparison with sequences retrieved from the National Center for Biotechnology Information database demonstrated that *E. coli isolate*s with integron sequences were detected in various food-producing animals and human hosts in environmental niches across Asia, Europe, and North America. These findings indicate the potential risk of ARG transmission between food-producing animals and humans by bacteria populations and provide useful baseline data for monitoring of ARGs.

**Supplementary Information:**

The online version contains supplementary material available at 10.1186/s12866-025-03794-y.

## Introduction

The increasing prevalence of antimicrobial resistance (AMR) poses a significant threat to global human health, a crisis emphasized by the World Health Organization in 2022 [1]. The imprudent utilization of antibiotics, including their overuse and misuse in human medicine, veterinary practice, and agriculture, leads to widespread AMR by accelerating the selection for AMR through underdosed antibiotic exposure, which promotes the acquisition of resistance genes (ARGs) and mobile genetic elements like integrons. Through horizontal gene transfer between different bacterial species, contributing to the emergence of multidrug-resistant (MDR) bacteria across various environments and hosts [2]. The spread of MDR bacteria in various environments and hosts including livestock, human populations, and agricultural, thereby establishing a complex chain of transmission that encompasses both the transmission from food-producing animals to the environment and the subsequent impact on human populations, as well as the bidirectional transmission between humans and animals through contact or the environment. Addressing the AMR within this chain is imperative and is currently pursued under the “One Health” framework [3]. Studies indicate a mortality rate associated with AMR, with approximately 700,000 deaths annually worldwide [4]. Notably, bacterial strains belonging to different species from food-producing animals, are responsible for an estimated 20% of drug-resistant infections in clinical human cases [5]. Among them, *Escherichia coli* coexists symbiotically within the gastrointestinal systems of humans and animals and harbors a vast array of ARGs. Thus, it serves as a dynamic reservoir for the dissemination of AMR. This organism not only harbors a vast array of ARGs but also serves as a dynamic hub for the dissemination of AMR [6].

Integrons, mobile genetic elements found predominantly in Gram-negative bacteria, notably *E. coli*, have garnered significant attention due to fundamental roles in propagating ARGs [7]. These genetic platforms have the capability to capture, exchange, and rearrange ARGs embedded within gene cassettes (GCs), thereby facilitating interbacterial transfer of AMR [8]. The typical structure of an integron encompasses three primary segments. Initiating this structure is the integrase gene (*intI*), which encodes a tyrosine-specific recombinase that orchestrates the processes of shuffling, integrating, or excising incoming GCs via site-specific, RecA-independent recombination. Subsequently, an integron-associated recombination site (*attI*) serves as the strategic locus for the insertion and recombination activities of GCs. The final segment comprises an integron-associated promoter (Pc) that is instrumental in regulation of the expression levels of GCs [9]. GCs are distinct mobile genetic elements that typically pair an open reading frame with a unique site-specific recombination locus known as *attC* to maintain the structural integrity and functional dynamics of the integron [10].

Integrons are categorized into five distinct classes based on the variability of the amino acid sequences of related integrases. However, only the classes I, II, and III have been associated with AMR [11]. Among these, class I integrons are the most widely distributed among *E. coli* from different sources and demonstrate connections with diverse insertion sequences or transposon families [12]. Despite the wide-ranging diversity of GCs associated with class I integrons, there is a frequent presence of GCs conferring resistance to trimethoprim (including the *dfr* genes), along with others providing resistance to streptomycin and spectinomycin (including the *aadA* genes). In addition, the sulfonamide resistance (*sul1*) gene is characteristically located at the 3’conserved segment (3’CS) [13]. In contrast to class I integrons, class II types are less prevalent and exclusively associated with Tn*7* transposons and related derivatives [14]. The amino acid sequences of integrases of class I and II integrons exhibit approximately 50% homology. A standard array of GCs in class II integrons, namely *dfrA1*-*sat1*/*2*-*aadA1*, has been identified, with the *sat1*/*2* gene imparting resistance to streptothricin [15]. Class III integrons, which have been identified in fewer than 10 bacterial species, typically harbor GCs that encode resistance mechanisms specifically targeting aminoglycosides [*aac(6’)*-*Ib*] and β-lactams (*bla*_OXA−256_, *bla*_GES_, *bla*_BEL_, and *bla*_IMP_) [16]. The diversity of reports on class I integrons underscores the need for intensified microbial surveillance to comprehensively understand the dissemination of AMR.

The previous evidence indicates that due to the high prevalence of class I integrons in *E. coli*, they can become vehicles for widespread dissemination of AMR in different niches and countries [17, 18]. Therefore, the aims of the present study were to assess the prevalence of class I integrons in *E. coli* isolates derived from food-producing animals and human patients, and to clarify potential linkages with interspecies dissemination of ARGs. For this purpose, *E. coli* isolates were collected from clinical specimens from patients in multiple hospital settings, along with samples from swine and poultry from various farms located throughout Zhejiang Province, China, from July 2019 to November 2022. The presence of class I integrons carried by *E. coli* isolates was assessed and potential correlations with AMR were investigated by comprehensive whole-genome sequencing. The results of this study should prove useful to elucidate the transmission mechanisms underlying AMR and to assess potential risks associated with gene transfer among bacteria across different hosts.

## Materials and methods

### Sampling, bacterial isolation and identification

**Fig. 1:**
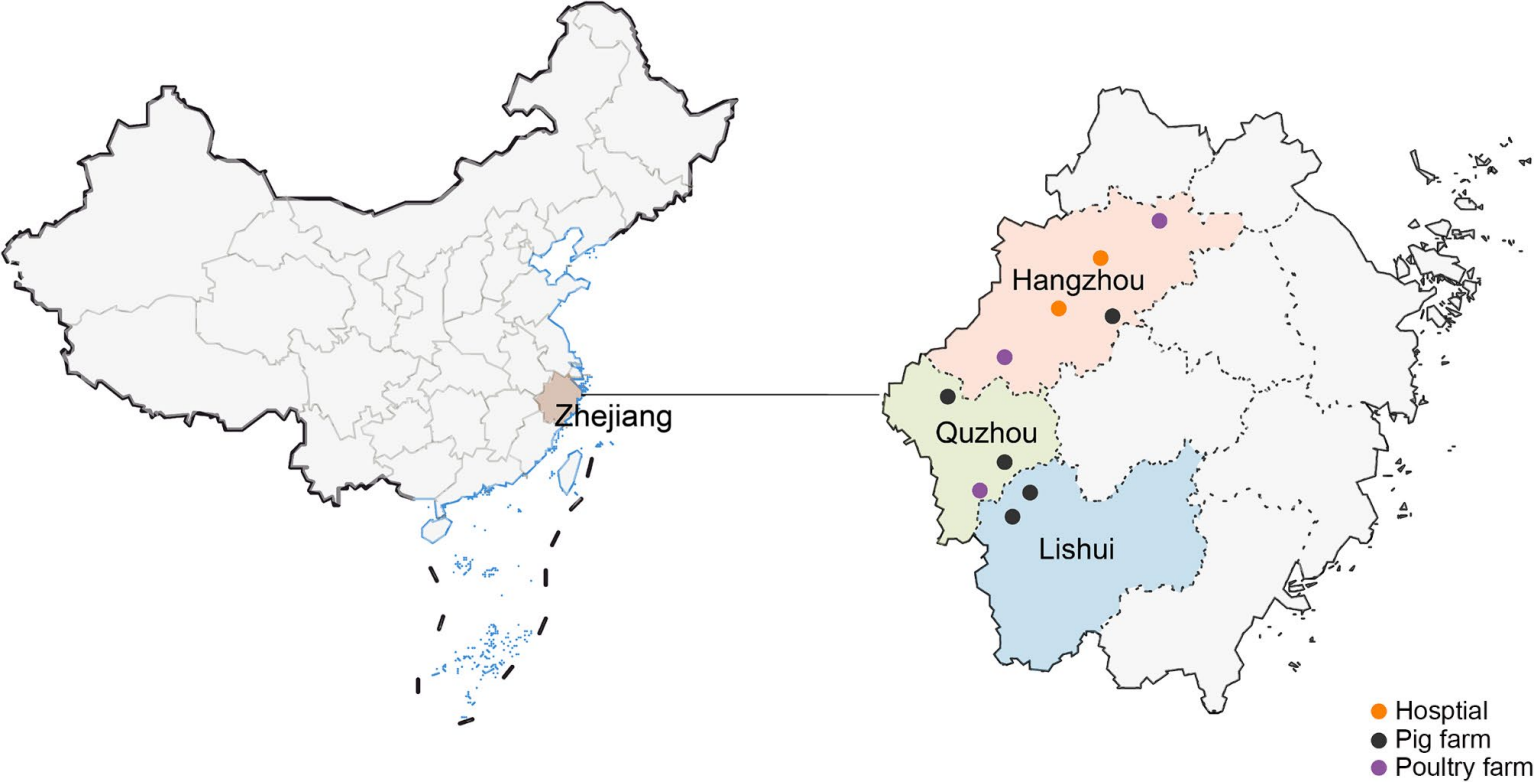
Geographical distribution of the sampling areas. The hospitals, pig and poultry farms are denoted with orange, black, and purple circles, respectively. The cities of Hangzhou, Quzhou, and Lishui are shaded in pink, green, and blue, respectively. Zhejiang Province is shaded in brown

Between June 2019 and November 2022, a total of 780 samples (415 anal swabs from pigs and 365 from different poultry species) were collected from various farms located in Hangzhou, Quzhou, and Lishui within Zhejiang Province (China) at unscheduled intervals to ensure randomness in sampling. The samples were preserved at 4 °C and transported to a designated laboratory under sterile conditions. All samples were processed within 24 h post-collection. As an initial culture stage, the anal swabs were grown in Luria-Bertani broth (Qingdao Hope Bio-Technology Co., Ltd., Qingdao, China). After overnight incubation, the cultured medium was streaked by inoculating loop onto eosin-methylene blue agar plates (Beijing Solarbio Science & Technology Co., Ltd., Beijing, China), and subsequently incubated at 37 °C for 16–18 h. Presumptive *E. coli* colonies were selected for further analysis. In addition, 113 *E. coli* isolates from patient samples collected from February to July 2021 were acquired from the Qingchun and Xiasha branches of Sir Run Shaw Hospital (Hangzhou, Zhejiang Province) (Fig. 1). A polymerase chain reaction (PCR) technique was employed to identify the *E. coli* isolates by *uid*A gene primers. As described previously [19], the PCR reaction system contained DNA template (1 µL), deoxynucleotide triphosphates (2 µL of 200 mM), 10× buffer (2.5 µL), primers (1 µL of each), Ex-Taq DNA polymerase (0.125 µL of 50 U). All the PCR reagents were purchased by Takara-Bio (Dalian, China). The PCR reaction procedure is as follows: 94 °C for 1 min; 30 cycles of 98 °C for 30 s, 55 °C for 30 s, 72 °C for 30 s; 72 °C for 10 min. The 5 µL PCR amplification product were analyzed by electrophoresis. Upon confirmation, the isolates were stored in 2-mL sterile tubes containing 50% glycerol at -80 °C for further analysis.

### Identification of the class I integrase gene intI1

Genomic DNA was extracted from all *E. coli* isolates using a bacterial genomic DNA extraction kit (General, Shanghai, China) in accordance with the manufacturer’s instructions. The primer pair *intI1*-F (5’-GGCTTCGTGATGCCTGCTT-3’) and *intI1*-R (5’-CATTCCTGGCCGTGGTTCT-3’) was designed to specifically amplify the target gene *intI1*. Each 25-µL PCR reaction volume included 2 µL of the DNA template, 12.5 µL of HotStarTaq^®^ Master Mix (Qiagen GmbH, Hilden, Germany), 1 µL of the forward primer (*intI1*-F), 1 µL of the reverse primer (*intI1*-R), and 8.5 µL of sterilized deionized water. The PCR cycling conditions included an initial denaturation step at 94 ^o^C for 1 min, followed by 35 cycles of denaturation at 94 ^o^C for 1 min, annealing at 55 ^o^C for 30 s, and extension at 72 ^o^C for 30 s, with a final extension step at 72 ^o^C for 10 min. The PCR amplification product were analyzed by electrophoresis.

### Antimicrobial susceptibility testing

Antimicrobial susceptibility of the *E. coli* isolates against 13 agents spanning seven distinct antimicrobial classes was assessed using the disk diffusion method, as established by the Clinical and Laboratory Standards Institute [20]. The antibiotic disks were obtained from Shanghai Yuanye Bio-Technology Co., Ltd. (Shanghai, China). The tested antibiotics included aminoglycosides (kanamycin [KAN], 30 µg; streptomycin [SM], 10 µg; neomycin [NEO], 30 µg), phenicols (chloramphenicol [CPL], 30 µg; florfenicol [FLO], 30 µg), β-lactams (ampicillin [AMP], 10 µg; meropenem [MRP], 10 µg), fluoroquinolones (enrofloxacin [ENR], 10 µg; ofloxacin [OFX], 5 µg), a lipopeptide (polymyxin B [PB], 300 µg), sulfonamides (sulfisoxazole [SIZ], 300 µg), tetracycline (TET, 30 µg) and trimethoprim (TMP, 5 µg). The *E. coli* isolates were incubated overnight at 37 °C and then diluted to 0.5 McFarland turbidity standard. Thus, 100 µL bacterial inoculum were plated on Mueller-Hinton agar (Qingdao Hope Bio-Technology Co., Ltd.) and incubated at 37 °C for 16–18 h. The inhibition zone diameters were measured to determine the level of resistance to the respective antibiotics according to the CLSI 2021. *E. coli* strain ATCC 25,922 was used for quality control. All *E. coli* isolates exhibiting resistance to three or more antibiotic classes were classified as MDR [19].

### Genome sequencing and data manipulation

The genomes of all *intI1*-positive *E. coli* isolates were sequenced using the HiSeq™ Sequencing System (Illumina, Inc., San Diego, CA, USA). DNA libraries were constructed from 0.2 µg of each DNA sample using the NEBNext^®^ Ultra™ DNA Library Prep Kit for Illumina (New England Biolabs, Ipswich, MA, USA) in accordance with the manufacturer’s guidelines. Each sample was assigned a unique index code. The DNA samples were fragmented by ultrasound into segments of approximately 350 bp, which were subjected to end repair by the addition of poly(A) tails and adapter ligation to ensure fidelity. Then, the segments were amplified by PCR to solidify the robustness of the library. The quality of the segments was assessed with a 5400 Fragment Analyzer system (Agilent Technologies, Inc., Santa Clara, CA, USA), while the quantity was measured by real-time PCR. Segments with a quality threshold < 20 were excluded from further analysis. Following pooling of the qualified segments, high-throughput sequencing was conducted with the HiSeq™ Sequencing System by Novogene Bioinformatics Technology Co., Ltd (Beijing, China). After a rigorous cleaning process, the raw sequences were assembled with CLC Genomics Workbench 12 software (CLC Bio, Aarhus, Denmark) to ensure precise and reliable sequence data for subsequent analyses.

### Genome sequence analysis

Multilocus sequence typing (MLST) was conducted using MLST 2 software (https://cge.ncbs.dtu.dk/services/MLST/). MLST allele sequence and profile data was obtained from PubMLST.org (https://pubmlst.org/*).* Subsequently, a minimal spanning tree of the isolates was generated with the GrapeTree tool (https://achtman-lab.github.io/GrapeTree/) [21]. The Power BI data and analytics reporting tool (https://www.process-science.com/power-bi/) was employed to elucidate the structural relationships among the isolates. Then, ResFinder 4.1 software (http://genepi.food.dtu.dk/resfinder) was used to identify AMR genes in the next-generation sequencing data [22]. A heatmap was generated using TBtools software [23] (https://github.com/CJ-Chen/TBtools-II) to visualize the distribution patterns of acquired AMR genes from diverse gene families across the genomes of individual isolates. Class I integrons were annotated with Integron_finder software [24] (https://github.com/gem-pasteur/Integron_Finder). Sequences retrieved from the National Center for Biotechnology Information (NCBI) database and aligned with the Basic Local Alignment Search Tool (http://blast.ncbi.nlm.nih.gov/Blast.cgi) were used to identify the AMR genes present on the GCs of integrons. Finally, Easyfig 2.2.5 software [25] (https://mjsull.github.io/Easyfig/) was employed for side-by-side comparative analysis of genetic landscapes.

### Statistical analysis

Within the parameters of this analysis, the scoring mechanism was straightforward: the presence of AMR genes was assigned a score of 1, while the absence of AMR genes was assigned a score of 0. The chi-square test was used to assess the relatedness of categorical variables. A probability (*p*) value < 0.05 was considered statistically significant.

## Results

### Sources of E. Coli isolates and detection of the class I integrase gene intI1

Of the 721 *E. coli* isolates examined in this study, 113 were from human patients from Sir Run Shaw Hospital and 608 were from food-producing animals raised on farms across Zhejiang Province (298 from pig anal swabs and 310 from poultry anal swabs) (Fig. 1).

Overall, 93 (12.90%) of the 721 *E. coli* isolates were positive for the class I integrase gene *intI1*. The prevalence of the *intI1*-positive isolates was 17.70% (20/113) in hospitalized patients, 17.45% (52/298) in pig samples, and only 6.77% (21/310) in poultry samples. Notably, *intI1* was considerably more common in pig samples and hospitalized patients than poultry samples (*p* < 0.05).

### Antimicrobial susceptibility of intI1-positive and -negative E. Coli isolates

**Fig. 2:**
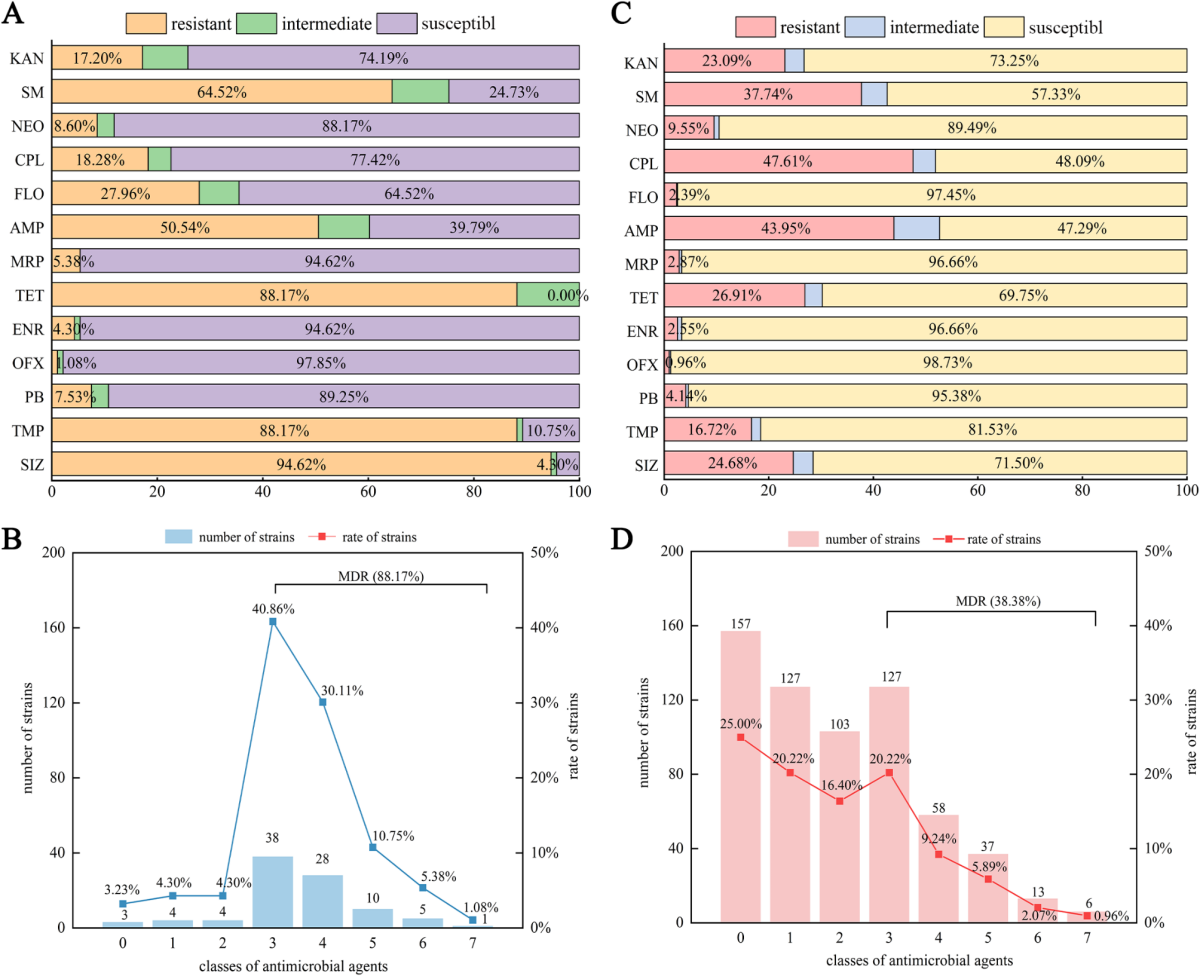
AMR profiles of *intI1*-positive and -negative *E. coli* isolates in this study. **(A)** AMR rates of 93 *intI1*-positive *E. coli* isolates. **(B)** The distribution of MDR strains among 93 *intI1*-positive *E. coli* isolates. **(C)** AMR rate of 628 *intI1*-negative *E. coli* isolates. **(D)** The distribution of MDR strains among 628 *intI1*- negative *E. coli* isolates. Kanamycin, KAN; streptomycin, SM; neomycin, NEO; chloramphenicol, CPL; florfenicol, FLO; ampicillin, AMP; meropenem, MRP; enrofloxacin, ENR; ofloxacin, OFX; polymyxin B, PB; sulfisoxazole, SIZ; tetracycline, TET; trimethoprim, TMP

The AMR profiles of 93 *intI1*-positive and 628 *intI1*-negative *E. coli* isolates are depicted in Fig. 2. Of the 93 *intI1*-positive *E. coli* isolates, 88 (94.62%), 82 (88.17%), 82 (88.17%), and 60 (64.52%) were resistant to SIZ, TMP, TET, and SM, respectively (Fig. 2A). In addition, 90 (96.77%) of the *intI1*-positive isolates were resistant to at least one antibiotic, with 82 (88.17%) categorized as MDR (Fig. 2B). Furthermore, 38 (40.86%) and 28 (30.11%) of the 93 *intI1*-positive isolates were resistant to three and four classes of antibiotics, respectively. A single *intI1*-positive *E. coli* isolate (1.08%) exhibited resistance across all seven antibiotic classes. Overall, there were 16 distinct AMR patterns, with resistance to KAN-CPL-TMP-SIZ emerging as the most prevalent at 33.33% (31/93) (Table S1).

Of the 628 *intI1*-negative *E. coli* isolates, 299 (47.61%), 276 (43.95%), 237 (37.74%), and 169 (26.91%) were resistant to CPL, AMP, SM, and TET, respectively (Fig. 2C). In addition, 471 (75.00%) of the 628 *intI1*-negative *E*. *coli* isolates exhibited resistance to at least one antibiotic class, with 241 (38.38%) categorized as MDR (Fig. 2D). Among these, 157 (25.00%), 127 (20.22%), and 127 (20.22%) were susceptible, one, and three classes of antibiotics, respectively. In addition, six *intI1*-negative *E. coli* isolates (0.96%) were resistant to all seven antibiotic classes. In total, 31 different AMR patterns were identified among the *intI1*-negative *E*. *coli* isolates, with CPL-AMP as the most common AMR pattern (9.39%, 59/628) (Table S2).

The MDR pattern was correlated with the presence of class I integrons (Table 1). The resistance rates to SM, CPL, FLO, TET, TMP and SIZ were significantly higher for isolates harboring class I integrons (*p* < 0.01).Also, the MDR rate was significantly higher for isolates containing class I integrons (*p* < 0.01). Meanwhile, there were no significant differences in the rates of resistance to KAN, NEO, AMP, MRP, ENR, OFX, and PB between isolates with and without class I integrons (*p* > 0.05).

**Table 1:** Association between antimicrobial resistance phenotypes of *intI1* positive or negative strains in 721 *E. Coli* isolates

Antibiotic	No. (%) of isolates		*p* value^a^
	intI1-positive strains (*n* = 93)	intI1-negative strains (*n* = 628)	
Kanamycin	16 (17.20)	145 (23.09)	0.2311
Streptomycin	60 (64.52)	237 (37.74)	< 0.0001*
Neomycin	8 (8.60)	60 (9.55)	0.4128
Chloramphenicol	17 (18.28)	299 (47.61)	< 0.0001*
Florfenicol	26 (27.96)	15 (2.39)	< 0.0001**
Ampicillin	47 (50.54)	276 (43.95)	0.2641
Meropenem	5 (5.38)	18 (2.87)	0.2030
Tetracyclines	82 (88.17)	169 (26.91)	< 0.0001**
Enrofloxacin	4 (4.30)	16 (2.55)	0.3116
Ofloxacin	1 (1.08)	6 (0.96)	0.8283
Polymyxin B	7 (7.53)	26 (4.14)	0.1771
Trimethoprim	82 (88.17)	105 (16.72)	< 0.0001**
Sulfisoxazole	88 (94.62)	155 (24.68)	< 0.0001**
Multidrug-resistant	82 (88.17)	241 (38.38)	< 0.0001**

^a^ Differences between *intI1* positive and negative strains were considered significant at *p* < 0.05* and extremely significant at *p* < 0.01**

### AMR gene patterns of intI1-positive E. Coli isolates

**Fig. 3:**
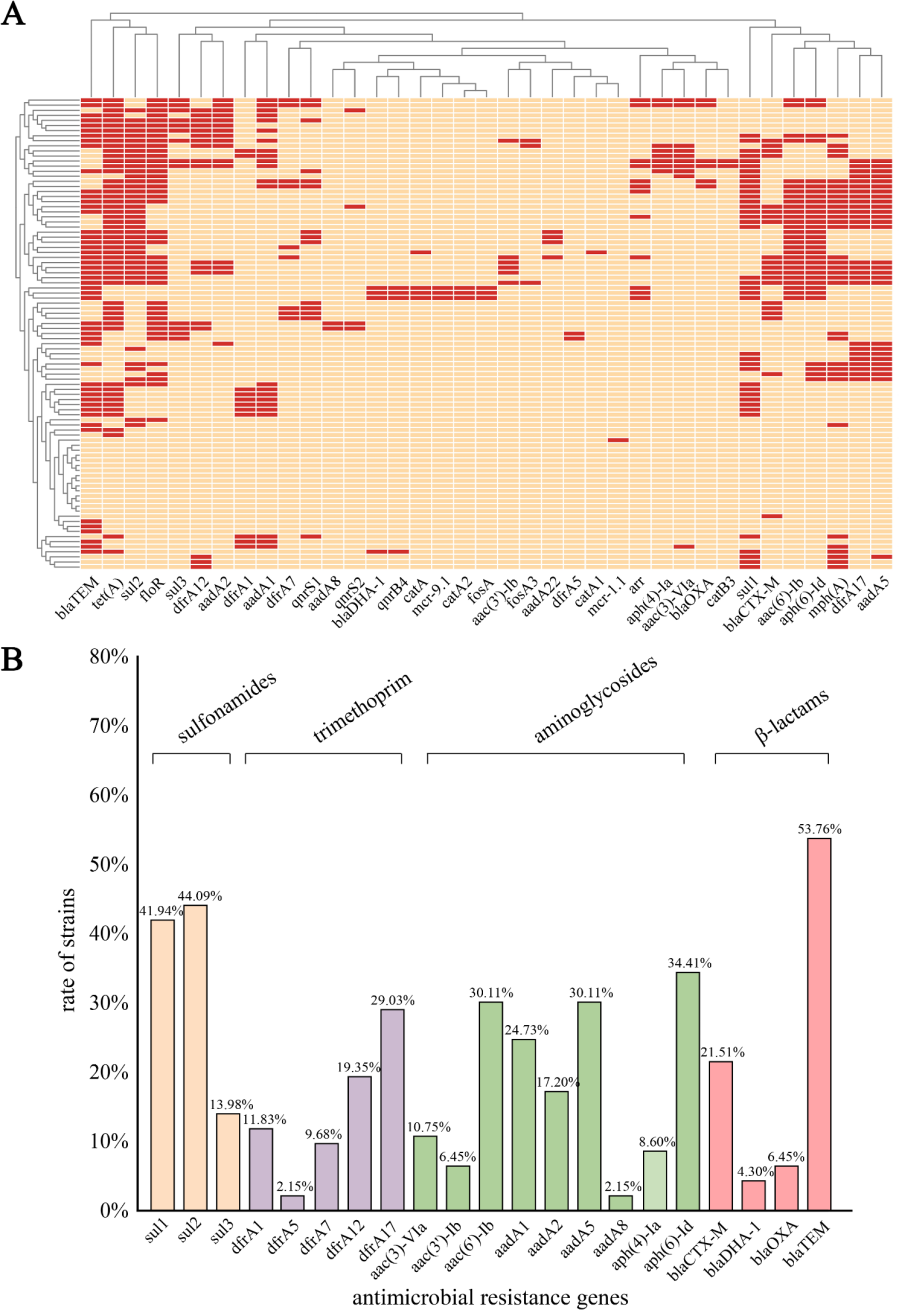
ARG patterns of *intI1*-positive *E. coli* isolates. **(A)** Distribution of acquired ARGs. The red and yellow colors indicate the existence and absence of ARGs, respectively. **(B)** Different classes of ARGs acquired by *intI1*-positive *E. coli* isolates

A diverse array of AMR genes was identified among the 93 *intI1*-positive *E*. *coli* isolates (Fig. 3A), which included genes conferring resistance to β-lactams (*bla*_CTX−M_, *bla*_DHA−1_, *bla*_OXA_, *bla*_TEM_), sulfonamides (*sul1*, *sul2*, *sul3*), tetracycline [*tet(A)*], aminoglycosides [*aac(3)-VIa*, *aac(3’)-Ib*, *aac(6’)-Ib*, *aadA1*, *aadA2*, *aadA5*, *aadA8*, *aph(4)-Ia*,* aph(6)-Id*], trimethoprim (*dfrA1*, *dfrA5*, *dfrA7*, *dfrA12*, *dfrA17*), fluoroquinolones (*qnrB4*, *qnrS1*, *qnrS2*), fosfomycins (*fosA*, *fosA3*), lipopeptides (*mcr-1.1*, *mcr-9.1*), macrolides [*mph(A)*], rifamycin (*arr*), and chloramphenicol (*floR*).

Prevalent resistance genes carried by the isolates included those conferring resistance to sulfonamides (*sul1* [41.94%], *sul2* [44.09%], *sul3* [13.98%]), trimethoprim (*dfrA1* [11.83%], *dfrA5* [2.15%], *dfrA7* [9.68%], *dfrA12* [19.35%], *dfrA17* [29.03%]), aminoglycosides (*aac(3)*-*VIa* [10.75%], *aac(3’)*-*Ib* [6.45%], *aac(6’)*-*Ib* [30.11%], *aadA1* [24.73%], *aadA2* [17.20%], *aadA5* [30.11%], *aadA8* [2.15%], *aph(4)*-*Ia* [8.60%], *aph(6)*-*Id* [34.41%]), and β-lactams (*bla*_CTX−M_ [21.51%], *bla*_DHA−1_ [4.30%], *bla*_OXA_ [6.45%], *bla*_TEM_ [53.76%]) (Fig. 3B).

### MLST analysis of intI1-positive E. Coli isolates

All 93 *intI1*-positive isolates were sequenced and subjected to MLST analysis. The draft genome length of these isolates was 4.46–5.37 Mb. In total, 39 sequence types (STs) with three (3/39) unknown STs were observed from all 93 isolates. Three novel ST profiles of seven *E. coli* isolates were identified with the EnteroBase online resource for analysis and visualization of genomic variation within enteric bacteria (https://enterobase.warwick.ac.uk/species/ecoli/allele_st_search), which included ST237112 (traced back to four distinct *E. coli* isolates derived from pig samples in Hangzhou), ST237113 (associated with two *E. coli* isolates from pig samples in Lishui), and ST237114 (linked to a single *E. coli* isolate from a pig sample collected in Hangzhou).

**Fig. 4:**
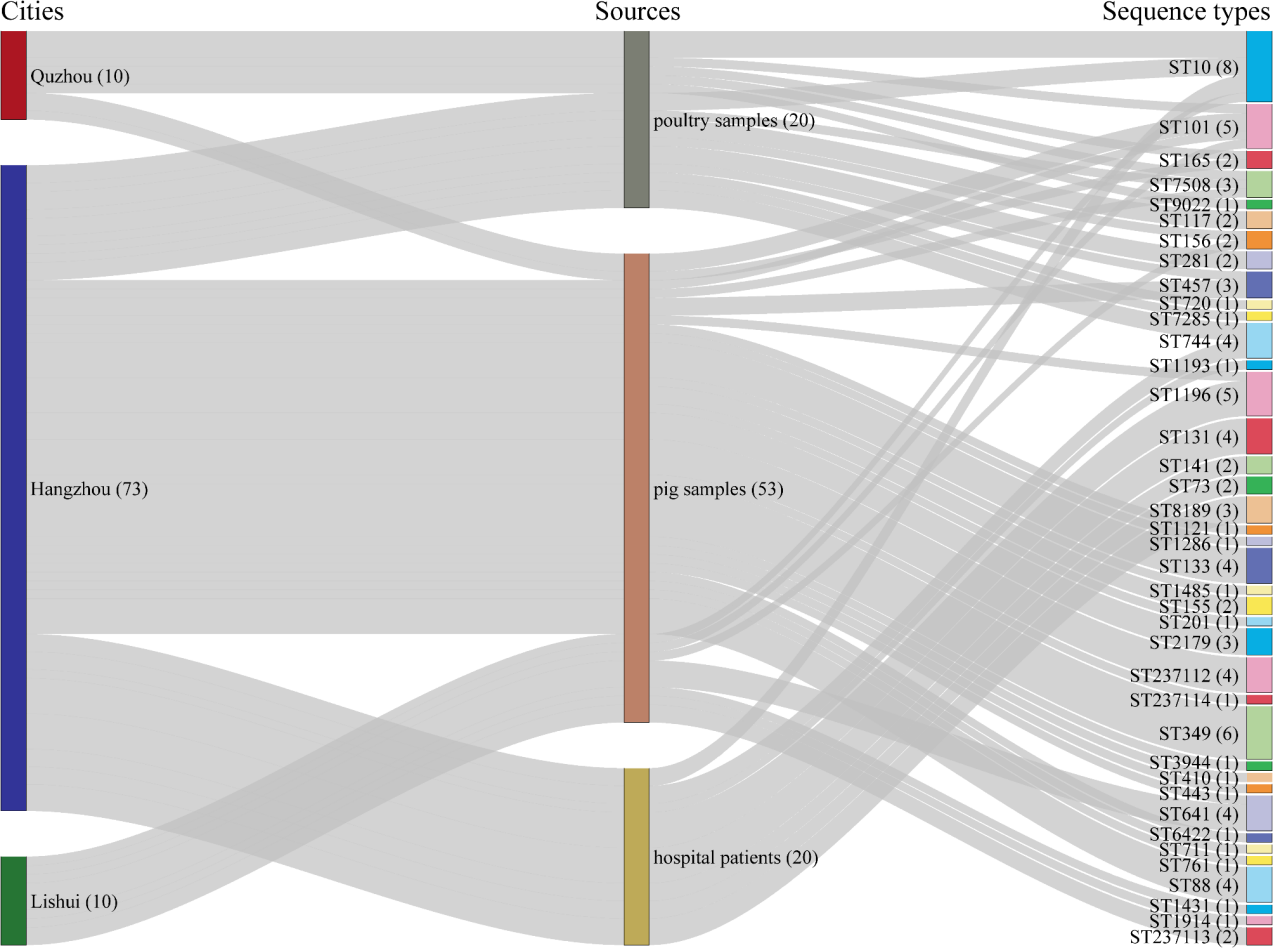
Prevalence of 93 *intI1*-positive *E. coli* isolates in this study. Sankey diagram combining the cities, sampling sources, and STs based on 93 *intI1*-positive *E. coli* isolates. The diameter of the line is proportional to the number of strains, which is also labeled with a number

Of the 93 *intI1*-positive isolates, ST10 (8.60%, 8/93) emerged as the most predominant ST. In addition, ST349, ST101 and ST1196, were each identified in 6.45% (6/93), 5.38% (5/93) and 5.38% (5/93) of the isolates. A detailed analysis of the 20 *intI1*-positive isolates derived from hospitalized patients revealed ST1196 and ST131 as the most common STs, each accounting for 20.00% (4/20). Meanwhile, ST349 was identified in six (11.32%) of the 53 *intI1*-positive *E*. *coli* isolates obtained from pig samples. Of the 20 *intI1*-positive isolates from poultry samples, ST10 was identified in five (25.00%). Notably, ST10 was present in isolates from hospitalized patients, as well as pig and poultry samples. ST101, ST156, ST165, ST457 and ST7508 were identified in both pig and poultry samples. Furthermore, ST1196 was common for pig samples and hospitalized patients, while ST744 was identified in both poultry samples and hospitalized patients. In summary, five distinct STs were observed across two or three different sources (Fig. 4).

### Characterization of class I integrons in E. Coli isolates

Of the 93 *intI1*-positive *E. coli* isolates, 59 (63.44%) harbored the classic class I integron, characterized by the *intI1* gene in the 5’CS and the *qacEΔ1* + *sul1* genes in the 3’CS. The *qacEΔ1* gene confers resistance to quaternary ammonium compounds, while the *sul1* gene confers resistance to sulfonamides. Overall, 33 isolates from pig samples, 17 from hospitalized patients, and nine from poultry samples harbored the classic class I integron genetic structure. Sources, STs, and arrangement of AMR GCs among the 59 *E. coli* isolates carrying classic class I integrons are listed in Tables 2 and Fig. S1. In total, six distinct AMR GCs were identified, with *dfrA17*-*aadA5* as the most prevalent at 33.40% (20/59), followed by *dfrA12*-*aadA2* (27.11%, 16/59), *dfrA1*-*aadA1* (22.03%, 13/59), *dfrA7* (8.47%, 5/59), *aac(6’)*-*Ib* (5.08%, 3/59), and *aadA1*-*aac(3)*-*VIa* (3.39%, 2/59). Remarkably, all 59 isolates with AMR GCs exhibited MDR. As shown in Table S3, 34 non-classic class I integrons lacked either GCs or the 3’CS region. Of these, 11 possessed only the *intI1* gene, five had GCs conferring resistance to trimethoprim, and 18 contained GCs associated with aminoglycoside resistance.

**Table 2:** Sources and sequence types of 59 classic class I integron-carrying *E. Coli* isolates and the arrangement of gene cassettes

Sources	Sequence types	Number (*n* = 59)	Gene cassette arrays
hospital patients	ST131	4	*dfrA17-aadA5* (*n* = 20)
	ST1193	1	
	ST141	2	
	ST8189	3	
pig samples	ST155	1	
	ST88	3	
	ST101	3	
	ST2179	1	
poultry samples	ST117	2	
hospital patients	ST1196	4	*dfrA12-aadA2* (*n* = 16)
pig samples	ST201	1	
	ST641	3	
	ST3944	1	
	ST7508	1	
	ST6422	1	
	ST2179	2	
	ST165	1	
poultry samples	ST7508	2	
hospital patients	ST10	2	*dfrA1-aadA1* (*n* = 13)
	ST73	1	
pig samples	ST156	1	
	ST349	6	
	ST1431	1	
poultry samples	ST7285	1	
	ST156	1	
pig samples	ST410	1	*dfrA7* (*n* = 5)
	ST101	1	
	ST133	2	
poultry sample	ST101	1	
pig samples	ST1914	1	*aac(6’)-Ib* (*n* = 3)
	ST9022	1	
poultry sample	ST720	1	
pig samples	ST1286	1	*aadA1-aac(3)-VIa* (*n* = 2)
	ST1485	1	

### Genetic environment of classic class I integrons

**Fig. 5:**
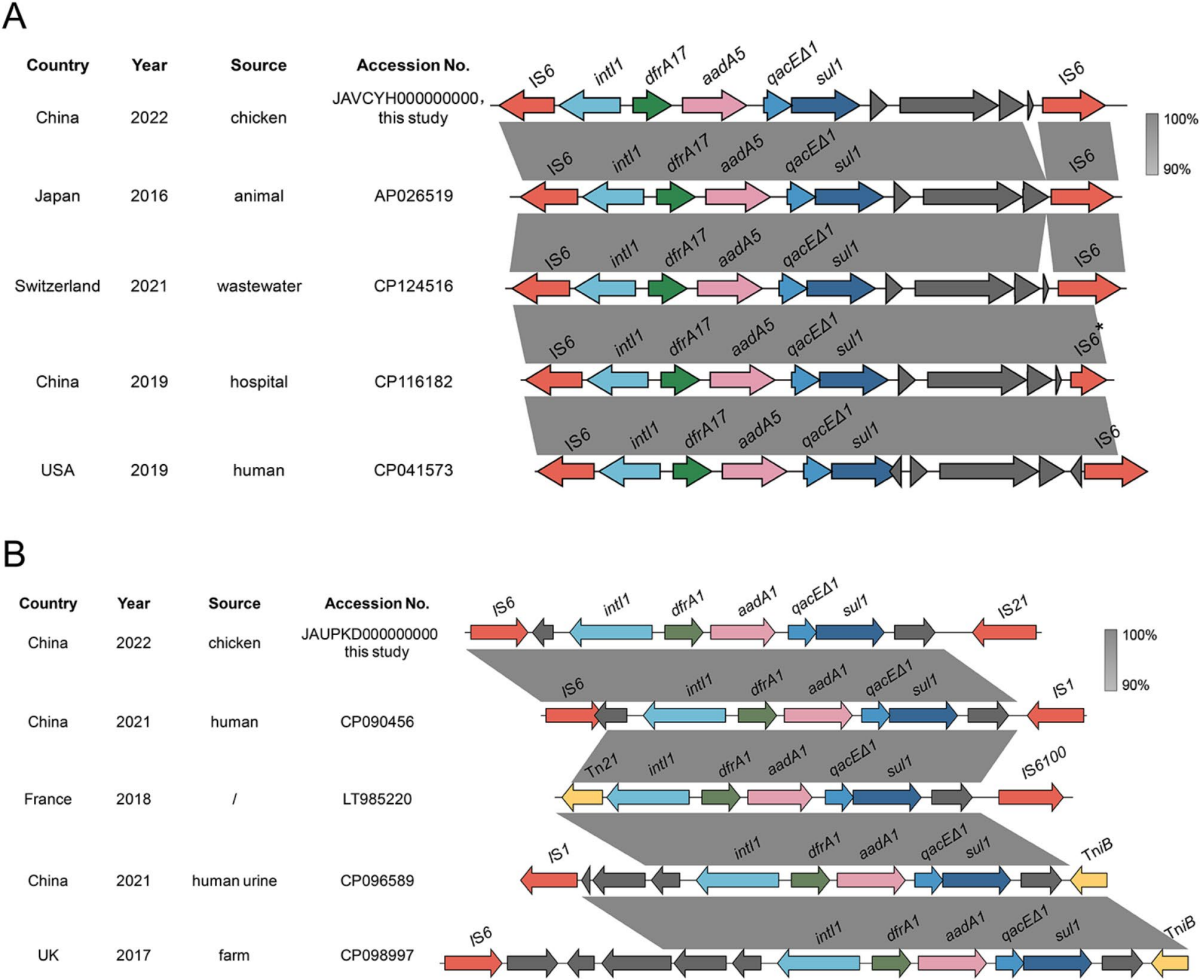
Genetic environment of class I integrons with the most frequent GCs in the genomes of *E. coli* isolates. **(A)** Genetic environment of class I integrons with the GC array *dfrA17-aadA5*. **(B)** Genetic environment of class I integrons with the GC array *dfrA1-aadA1.* Arrows indicate the direction of transcription. Regions of > 90% homology are shaded in gray. Gene families are differentiated by different colors. * means missing the C-terminus

Characterization of the genomic contexts of 59 classic class I integrons revealed the presence of six insertion sequences (IS*1*, IS*6*, IS*21*, IS*91*, IS*110*, and IS*256*) and one transposon (Tn*3*) adjacent to the integrons (Table S4). A comprehensive analysis to elucidate potential transmission pathways of these integrons both in the context of this study and globally found that all 20 integron sequences associated with the predominant GCs (*dfrA17*-*aadA5*) identified in this study were bordered by IS*6* elements. A comparative sequence analysis using the NCBI database was conducted focusing on the “*intI1-dfrA17-aadA5-qacEΔ1-sul1*” class I integron sequence. Four *E. coli* isolates exhibiting the highest sequence homology were selected for in-depth comparisons. Notably, strains containing this specific integron sequence were documented in poultry (this study, 2022), an unspecified animal in Japan (2016), wastewater in Switzerland (2021), a Chinese hospital, and a human subject in the USA (both 2019). Each of these isolates harbored integrons flanked by IS*6* elements (Fig. 5A). Furthermore, integron sequences with the second most common gene cassettes (*dfrA1*-*aadA1*) identified in this study was associated with IS*1*, IS*6*, IS*21*, or Tn*3* elements. Similar sequences from the NCBI database were also affiliated with IS*6100*, Tn*21*, and Tn*iB* elements (Fig. 5B). These integron sequences were identified in various environments, including poultry, humans, and farms, and were geographically dispersed, with instances recorded in China, France, and the UK over different periods.

As shown in Fig. S2, further analysis was conducted on other frequently observed integron sequences, namely “IS*6-intI1-dfrA12-aadA2-qacEΔ1-sul1-*IS*91*” (Fig. S2A), “Tn*3-intI1-dfrA7-qacEΔ1-sul1-*IS*21*” (Fig. S2B), “IS*6-intI1-aac(6’)-Ib-qacEΔ1-sul1-*IS*6*” (Fig. S2C), and “Tn*3-intI1-aadA1-aac(3)-VIa-*IS*91-*IS*256-qacEΔ1-sul1-*IS*110*” (Fig. S2D). These sequences were compared with others retrieved from the NCBI database. Strains harboring these integron configurations were bordered by an assortment of insertion sequences and transposons. These *E. coli* isolates were identified in various host organisms and environmental samples, including animals and meat products (e.g., pigs, poultry, turkeys, veal, other avian species), blood and urine samples collected from hospitalized patients and the community, and environmental sources (wastewater and surface water). Geographic analysis revealed a wide distribution of these strains across continents, occurring in Asia (China and Singapore), Europe (Switzerland, Norway, Italy, France, and Spain), and North America (USA).

## Discussion

The global challenge posed by AMR needs embracing of the “One-Health” strategy, an approach advocating for the interconnectedness of human and animal healthcare sectors and the environment [26, 27, 28, 29]. Class I integrons have been identified as crucial vehicles in the transmission of ARGs, but also roles as molecular sentinels, thus offering comprehensive and dependable assessment of the propagation of AMR [24, 25, 26, 27, 28, 29, 30]. Moreover, *E. coli* is an important target for surveillance of AMR due to its wide diversity of hosts and propensity for the acquisition of ARGs through diverse genetic elements [19]. In the present study, 12.90% of the *E. coli* isolates carried class I integrons. Notably, the detection frequency of the *intI1* gene in pig and human samples was higher than that of poultry samples. This observation aligns with findings from various geographical locations, underscoring the ubiquity of class I integrons. In previous reports, the prevalence of class I integrons was 63.33% in *E. coli* isolates from human samples in Turkey [31], 52% in human-derived isolates in Iran [32], and 49% in poultry-derived isolates in Algeria [33]. In addition, the prevalence of class I integrons were reportedly lower in Thailand, with 11.5% and 10.8% of pig and poultry *E. coli* isolates, respectively [34]. These data collectively highlight that prevalence of class I integrons in *E. coli* are consistently above 10% in food-producing animals [31–32].

The AMR phenotypes revealed differences between *E. coli* isolates with and without class I integrons. Isolates positive for the *intI1* gene exhibit a significant increase in resistance rates, particularly to florfenicol, tetracycline, trimethoprim, sulfisoxazole, and streptomycin, along with a greater likelihood for MDR. Prior investigations elucidated that the assimilation of class I integrons predisposes bacteria to an influx of exogenous genetic elements, thereby fortifying AMR defenses and facilitating MDR development [35]. Nonetheless, isolates with class I integrons exhibit significantly diminished resistance to chloramphenicol as compared to those devoid of such mobile genetic elements. This phenomenon indicates the intricate dynamics governing AMR and advocates for an in-depth exploration of the underlying mechanisms.

A comparative analysis of the AMR phenotypes and genotypes of *intI1*-positive *E. coli* isolates determined similar resistance against sulfonamides and trimethoprim likely due to the close association of GCs within class I integrons. However, the resistance profiles of aminoglycosides and β-lactams were identified inconsistent with the genotypes. The aminoglycoside- and β-lactam-specific ARGs in these isolates may be unexpressed, contributing to the discrepancy [36]. Furthermore, several robust correlations between the AMR phenotypes and genotypes of *E. coli* suggest that certain resistance profiles were determined by genotypes [37]. Conversely, anomalies were observed wherein certain isolates exhibited resistance phenotypes absent of corresponding ARGs. This phenomenon may be related to the ARG genetic linkage, co-selection with different ARGs and some unknown mechanisms so far [38].

In this study, GCs encoding *dfr* and *aadA* genes emerged as the predominant constituents of the class I integrons of *E. coli*, a phenomenon echoed in diverse Gram-negative bacteria, encompassing species like *Aeromonas* in aquatic environments, *Salmonella* in poultry, and *Klebsiella pneumoniae* in human hosts [39, 40, 41]. The stability of these two GCs is noteworthy, typically occupying the prime locus following *intI1*, thereby facilitating global dissemination with class I integrons, inclusive in absence of selective pressures [42]. Furthermore, *aac(6’)*-*Ib* and *aac(3)*-*VIa* encoding GCs were present in three strains, aligning with previous reports of GCs as frequent constituents within class I integrons in divergent regions, such as China and the USA [43–44]. Interestingly, non-standard class I integrons detected in 38 isolates were characterized by the absence of the conventional 3’ CS (*qacEΔ1* + *sul1*), linkages with *sul2* or *sul3* genes within the 3’ CS, or only containing the integrase gene *intI1*. Previous reports confirmed the widespread presence of non-classical class I integrons in bacterial populations from humans and food-producing animals [45–46].

Class I integrons with related GCs were probably spread by vertical transfer between different reservoirs of human and animal origin [47–48]. Insights into the regional dispersion patterns and phylogenetic correlations of individual isolates can be discerned through molecular techniques like MLST [49]. Also, the ST designations can be useful to characterize and monitor vertical transmission of disease-causing and AMR lineages of bacteria [50–51]. In the present analysis, among the 93 *intI1*-positive *E*. *coli* isolates, 39 distinct STs were identified, including three previously unidentified STs, demonstrating considerable heterogeneity among *E. coli* isolates possessing class I integrons. Notably, 8.6% of these isolates were classified as ST10, and detected in pigs, poultry, and human populations. Previous studies have identified this ST in various sources, including food-producing animals and retail meats, extending to human samples in multiple countries like Germany, Denmark, Ireland, and Spain, often associated to MDR [52, 53, 54]. Furthermore, ST131 has garnered attention as a prevalent lineage among MDR *E. coli* isolates [55]. In this research, ST131 emerged as a dominant ST in human subjects, but were non-existent in food-producing animals. This distribution pattern of various STs in *E. coli* with class I integrons suggests the potential of clonal propagation. The global spread of certain high-risk *E. coli* clones, especially ST10 and ST131, known for carrying MDR determinants and mobile genetic elements, poses a significant threat to both veterinary and human healthcare [52, 53, 54, 55]. This situation necessitates rigorous monitoring strategies, aligning with the “One Health” approach [56]. These findings add to the global database of *E. coli* STs associated with class I integrons and provides a basis for tracking the spread of these resistant strains.

In the present study, gene sequence alignment identified fragments related to class I integrons and revealed the presence of insertion sequences and transposons from multiple families located at various sites. Members of the IS*6* family, particularly noted for associations with GCs of the *dfrA17*-*aadA5* combination, are critical contributors to the spread of resistance markers in Gram-negative bacteria [57]. Moreover, members of the Tn*3* family emerged as prevalent components of the transposons of the *E. coli* isolates, consistent with previous reports [58]. Additional insertion sequence and transposon families, including IS*1*, IS*21*, IS*91*, IS*110*, IS*256*, IS6*100*, Tn*iB*, and Tn*1696*, theoretically capable of mobilization of class I were also identified [59]. These observations suggest worldwide spread of class I integrons among human and food-producing animal populations, highlighting the need for enhanced surveillance and control strategies. The findings expand the current database of mobile genetic elements associated with integrons, contributing to the understanding of their role in AMR gene dissemination. In the meanwhile, the study is provides a detailed analysis of class I integrons in *E. coli* isolates from both humans and food-producing animals in a rural area of Zhejiang Province, China. This geographical focus adds to the global understanding of AMR dynamics in a region, broadening the scientific knowledge on integron distribution.

Plasmids, particularly conjugative plasmids, like class I integrons, play a crucial role in the horizontal transfer of ARGs and are key contributors to the spread of AMR. While our study provides valuable insights into the prevalence and characteristics of class I integrons in *E. coli* isolates from humans and food-producing animals, it is important to provide a more comprehensive understanding of the mechanisms underlying the spread of plasmid and associated resistance genes among different reservoirs of human and animal origin in the next further studies.

## Conclusions

In this study carried out in a rural area of Zhejiang Province, China, from 2019 to 2022, we determined a relatively low prevalence of class I integrons of 12.9% among *E. coli* from humans and food-producing animals. Among the *intI1*-positive *E. coli* isolates, most of them possessed classic class I integrons with six distinct GC arrangements. MLST analysis showed a high heterogenicity, inclusive of three previously unidentified STs. Notably, ST10 emerged as the predominant genotype in samples from hospitalized patients, pigs, and poultry. Genomic analysis further identified six insertion sequences (IS*1*, IS*6*, IS*21*, IS*91*, IS*110*, and IS*256*) and one transposon (Tn*3*) in proximity to the integrons. A comprehensive exploration of the NCBI database affirmed the presence of a diverse array of insertion sequences and transposons within the integron sequences of *E. coli* isolates from animals, meat products, humans, and various environmental samples across Asia, Europe, and North America. These findings collectively offer insights into the potential risks associated with the transmission of class I integrons among bacteria, thus underscoring global implications for both humans and food-producing animals.

## Electronic supplementary material

Below is the link to the electronic supplementary material.

Supplementary Material 1

Supplementary Material 2

Supplementary Material 3

Supplementary Material 4

Supplementary Material 5

Supplementary Material 6

## Data Availability

All contiguous sequences have been submitted to GenBank and assigned accession numbers under BioProject PRJNA1171180, PRJNA1004883, PRJNA998200, PRJNA1007277, PRJNA998195, PRJNA998198 and PRJNA1004768. Other datasets used and/ or analysed during the current study are available from the corresponding author on reasonable request.
