# Supplementary figures and images for: Prevalence and characterization of class I integrons in multidrug-resistant Escherichia coli isolates from humans and food-producing animals in Zhejiang Province, China
Source: BMC Microbiol. 2025 Feb 15;25:76. doi: 10.1186/s12866-025-03794-y (PMC11830211; doi:10.1186/s12866-025-03794-y)

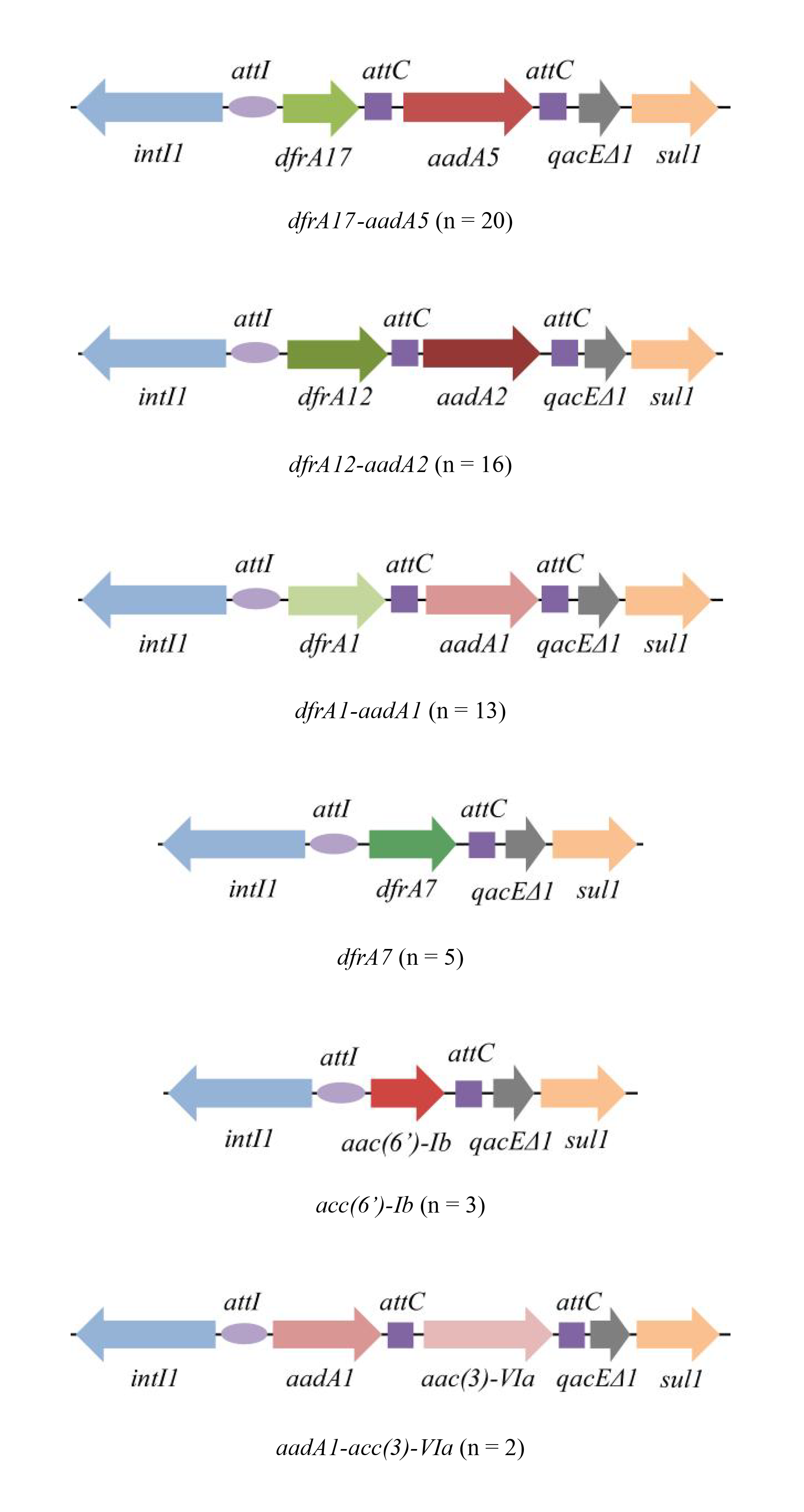

Supplement: Supplementary file 1 — Supplementary Material 1 [file 12866_2025_3794_MOESM1_ESM.tif]

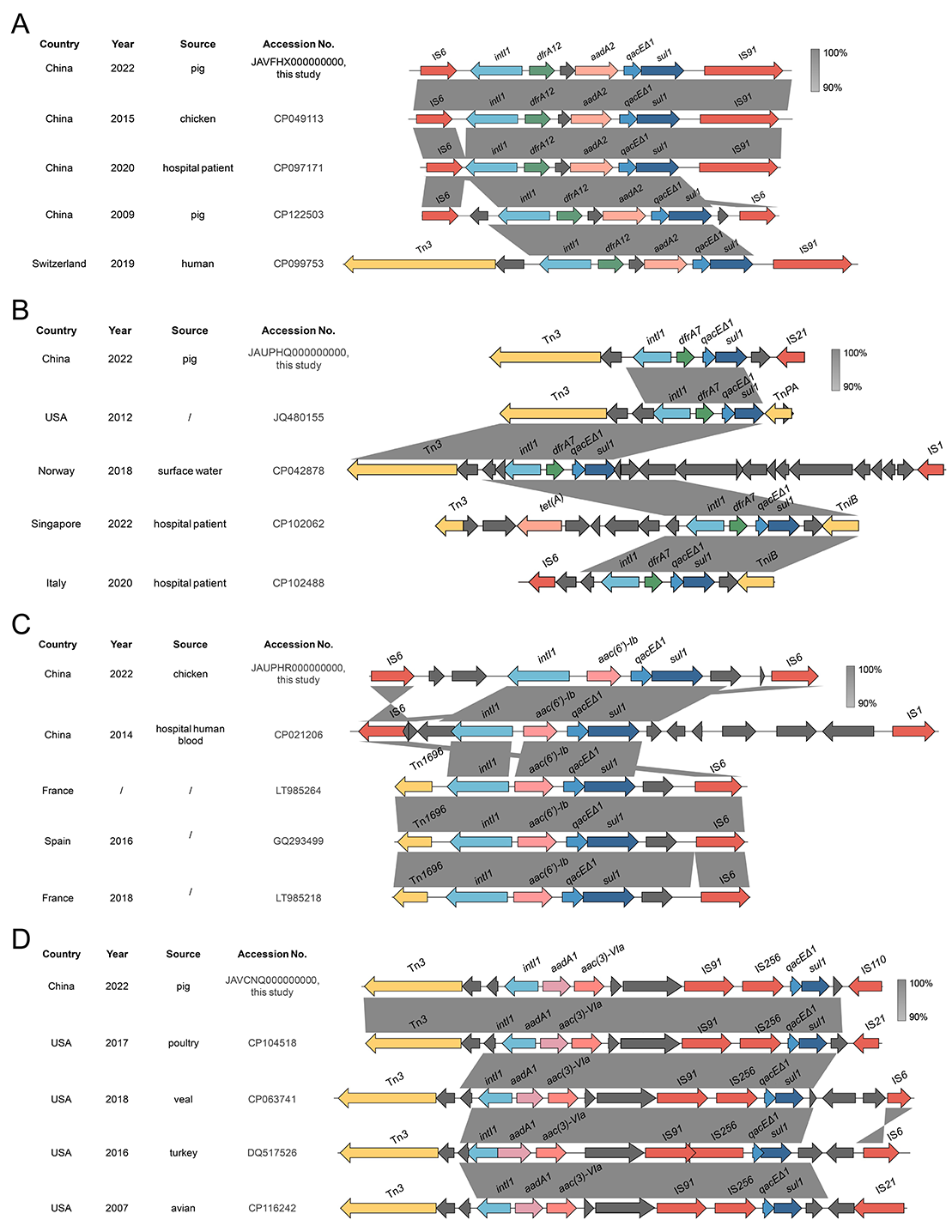

Supplement: Supplementary file 2 — Supplementary Material 2 [file 12866_2025_3794_MOESM2_ESM.tif]
