# Supplementary material for: Prevalence and characterization of class I integrons in multidrug-resistant Escherichia coli isolates from humans and food-producing animals in Zhejiang Province, China
Source: BMC Microbiol. 2025 Feb 15;25:76. doi: 10.1186/s12866-025-03794-y (PMC11830211; doi:10.1186/s12866-025-03794-y)
Supplement: Supplementary file 3 — Supplementary Material 3 [file 12866_2025_3794_MOESM3_ESM.docx]

**Table S1** Antimicrobial resistance patterns of 93 *intI1*-positive *E. coli* isolates

| **Antibiotic classes** | **Antibiotic numbers** | **Antimicrobial resistance patterns** | **Isolate number** | **Isolation rate**  **(%)** |
| --- | --- | --- | --- | --- |
| 0 | 0 | / | 3 | 3.23 |
| 1 | 1 | SIZ | 2 | 2.15 |
| 1 | 1 | AMP | 1 | 1.08 |
| 1 | 1 | TET | 1 | 1.08 |
| 2 | 2 | AMP-SIZ | 2 | 2.15 |
| 2 | 3 | TET-TMP-SIZ | 2 | 2.15 |
| 3 | 4 | AMP-TET-TMP-SIZ | 5 | 5.38 |
| 3 | 5 | KAN-SM-AMP-TMP-SIZ | 2 | 2.15 |
| 3 | 4 | KAN-CPL-TMP-SIZ | 31 | 33.33 |
| 4 | 6 | SM-AMP-FLO-TET-TMP-SIZ | 23 | 24.72 |
| 4 | 6 | SM-FLO-MRP-TET-TMP-SIZ | 3 | 3.23 |
| 4 | 6 | KAN-SM-MRP-CPL-TMP-SIZ | 2 | 2.15 |
| 5 | 6 | KAN-SM-CPL-ENR-PB-SIZ | 2 | 2.15 |
| 5 | 7 | KAN-SM-CPL-AMP-TET-TMP-SIZ | 8 | 8.60 |
| 6 | 8 | KAN-SM-CPL-AMP-TET-PB-TMP-SIZ | 5 | 5.38 |
| 7 | 9 | KAN-SM-CPL-AMP-TET-OFX-PB-TMP-SIZ | 1 | 1.08 |

Kanamycin, KAN; streptomycin, SM; neomycin, NEO; chloramphenicol, CPL; florfenicol, FLO; ampicillin, AMP; meropenem, MRP; enrofloxacin, ENR; ofloxacin, OFX; polymyxin B, PB; sulfisoxazole, SIZ; tetracycline, TET; trimethoprim, TMP.
