# Supplementary material for: Prevalence and characterization of class I integrons in multidrug-resistant Escherichia coli isolates from humans and food-producing animals in Zhejiang Province, China
Source: BMC Microbiol. 2025 Feb 15;25:76. doi: 10.1186/s12866-025-03794-y (PMC11830211; doi:10.1186/s12866-025-03794-y)
Supplement: Supplementary file 4 — Supplementary Material 4 [file 12866_2025_3794_MOESM4_ESM.docx]

**Table S2** Antimicrobial resistance patterns of 628 *intI1*-negative *E. coli* isolates

| **Antibiotic classes** | **Antibiotic**  **numbers** | **Antimicrobial resistance patterns** | **Isolate number** | **Isolation rate**  **(%)** |
| --- | --- | --- | --- | --- |
| 0 | 0 | / | 157 | 25.00 |
| 1 | 1 | AMP | 37 | 5.89 |
| 1 | 1 | SM | 29 | 4.62 |
| 1 | 2 | TMP-SIZ | 17 | 2.71 |
| 1 | 1 | SIZ | 17 | 2.71 |
| 1 | 1 | CPL | 16 | 2.55 |
| 1 | 1 | TET | 11 | 1.75 |
| 2 | 2 | CPL-AMP | 59 | 9.39 |
| 2 | 4 | SM-NEO-TMP-SIZ | 30 | 4.78 |
| 2 | 3 | CPL-TET-SIZ | 8 | 1.27 |
| 2 | 3 | TET- TMP-SIZ | 6 | 0.96 |
| 3 | 3 | KAN-CPL-AMP | 44 | 7.01 |
| 3 | 4 | KAN-SM-CPL-TET | 41 | 6.53 |
| 3 | 4 | KAN-SM-CPL-AMP | 13 | 2.07 |
| 3 | 3 | SM-CPL-TET | 10 | 1.59 |
| 3 | 4 | KAN-SM-AMP-TET | 9 | 1.43 |
| 3 | 4 | SM-TET-TMP-SIZ | 6 | 0.96 |
| 3 | 4 | KAN-CPL-TMP-SIZ | 4 | 0.64 |
| 4 | 5 | SM-NEO-CPL-AMP-TET | 21 | 3.34 |
| 4 | 4 | KAN-SM-CPL-AMP | 17 | 2.71 |
| 4 | 5 | SM-CPL-FLO-AMP-ENR | 9 | 1.43 |
| 4 | 5 | CPL-AMP-TET-TMP-SIZ | 5 | 0.80 |
| 4 | 6 | SM-AMP-FLO-TET-TMP-SIZ | 6 | 0.96 |
| 5 | 7 | SM-CPL-AMP-MRP-TET-TMP-SIZ | 11 | 1.75 |
| 5 | 6 | KAN-SM-CPL-AMP-PB-SIZ | 10 | 1.59 |
| 5 | 6 | SM-CPL-AMP-TET-TMP-SIZ | 9 | 1.43 |
| 5 | 7 | SM-AMP-MRP-TET-PB-TMP-SIZ | 4 | 0.64 |
| 5 | 6 | NEO-CPL-AMP-MRP-TET-SIZ | 3 | 0.48 |
| 6 | 7 | KAN-CPL-AMP-TET-ENR-TMP-SIZ | 7 | 1.11 |
| 6 | 6 | SM-CPL-AMP-TET-PB-SIZ | 6 | 0.96 |
| 7 | 8 | SM-NEO-CPL-AMP-TET-OFX-PB-TMP-SIZ | 6 | 0.96 |

Kanamycin, KAN; streptomycin, SM; neomycin, NEO; chloramphenicol, CPL; florfenicol, FLO; ampicillin, AMP; meropenem, MRP; enrofloxacin, ENR; ofloxacin, OFX; polymyxin B, PB; sulfisoxazole, SIZ; tetracycline, TET; trimethoprim, TMP.
