# Supplementary material for: Prevalence and characterization of class I integrons in multidrug-resistant Escherichia coli isolates from humans and food-producing animals in Zhejiang Province, China
Source: BMC Microbiol. 2025 Feb 15;25:76. doi: 10.1186/s12866-025-03794-y (PMC11830211; doi:10.1186/s12866-025-03794-y)
Supplement: Supplementary file 5 — Supplementary Material 5 [file 12866_2025_3794_MOESM5_ESM.docx]

**Table S4** The gene cassette arrays, sequence types and integron sequences with flanking sequences of 59 classic class I integrons

| Sample ID | Gene cassette arrays | Sequence types | Integron sequences with flanking sequences |
| --- | --- | --- | --- |
| SYF21_1 | *dfrA17-aadA5* | ST131 | IS*6-intI1-dfrA17-aadA5-qacEΔ1-sul1-*IS*6* |
| SYF32_1 | *dfrA17-aadA5* | ST131 | IS*6-intI1-dfrA17-aadA5 -qacEΔ1-sul1-*IS*6* |
| SYF331_1 | *dfrA17-aadA5* | ST131 | IS*6-intI1-dfrA17-aadA5 -qacEΔ1-sul1-*IS*6* |
| SYF367_1 | *dfrA17-aadA5* | ST131 | IS*6-intI1-dfrA17-aadA5-qacEΔ1-sul1-*IS*6* |
| SYF18_1 | *dfrA17-aadA5* | ST1193 | IS*6-intI1-dfrA17-aadA5 -qacEΔ1-sul1-*IS*6* |
| SYF336_1 | *dfrA17-aadA5* | ST141 | IS*6-intI1-dfrA17-aadA5 -qacEΔ1-sul1-*IS*6* |
| SYF161_1 | *dfrA17-aadA5* | ST141 | IS*6-intI1-dfrA17-aadA5 -qacEΔ1-sul1-*IS*6* |
| SYF173_1 | *dfrA17-aadA5* | ST8189 | IS*6-intI1-dfrA17-aadA5 -qacEΔ1-sul1-*IS*6* |
| SYF318_1 | *dfrA17-aadA5* | ST8189 | IS*6-intI1-dfrA17-aadA5 -qacEΔ1-sul1-*IS*6* |
| SYF321_1 | *dfrA17-aadA5* | ST8189 | IS*6-intI1-dfrA17-aadA5 -qacEΔ1-sul1-*IS*6* |
| HZZ10_1 | *dfrA17-aadA5* | ST155 | IS*6-intI1-dfrA17-aadA5 -qacEΔ1-sul1-*IS*6* |
| HZZ106 | *dfrA17-aadA5* | ST88 | IS*6-intI1-dfrA17-aadA5 -qacEΔ1-sul1-*IS*6* |
| HZZ3-8 | *dfrA17-aadA5* | ST88 | IS*6-intI1-dfrA17-aadA5 -qacEΔ1-sul1-*IS*6* |
| HZZ3-12 | *dfrA17-aadA5* | ST88 | IS*6-intI1-dfrA17-aadA5 -qacEΔ1-sul1-*IS*6* |
| LSSZ07_1 | *dfrA17-aadA5* | ST101 | IS*6-intI1-dfrA17-aadA5 -qacEΔ1-sul1-*IS*6* |
| QZZ01_1 | *dfrA17-aadA5* | ST101 | IS*6-intI1-dfrA17-aadA5 -qacEΔ1-sul1-*IS*6* |
| QZZ03_1 | *dfrA17-aadA5* | ST101 | IS*6-intI1-dfrA17-aadA5 -qacEΔ1-sul1-*IS*6* |
| HZZ7-4_1 | *dfrA17-aadA5* | ST2179 | IS*6-intI1-dfrA17-aadA5 -qacEΔ1-sul1-*IS*6* |
| HZDJ102_1 | *dfrA17-aadA5* | ST117 | IS*6-intI1-dfrA17-aadA5 -qacEΔ1-sul1-*IS*6* |
| HZRJ110_1 | *dfrA17-aadA5* | ST117 | IS*6-intI1-dfrA17-aadA5 -qacEΔ1-sul1-*IS*6* |
| SYF317_1 | *dfrA1-aadA1* | ST10 | IS*6-intI1-dfrA1-aadA1 -qacEΔ1-sul1-*IS*6* |
| SYF60_1 | *dfrA1-aadA1* | ST10 | IS*6-intI1-dfrA1-aadA1 -qacEΔ1-sul1-*IS*6* |
| SYF389_1 | *dfrA1-aadA1* | ST73 | IS*6-intI1-dfrA1-aadA1-qacEΔ1-sul1-*Tn*3* |
| LSSZ53 | *dfrA1-aadA1* | ST156 | IS*6-intI1-dfrA1-aadA1-qacEΔ1-sul1-*Tn*3* |
| HZDJ124_1 | *dfrA1-aadA1* | ST156 | IS*6-intI1-dfrA1-aadA1-qacEΔ1-sul1-*Tn*3* |
| HZZ8-9 | *dfrA1-aadA1* | ST349 | IS*6-intI1-dfrA1-aadA1-qacEΔ1-sul1-*IS*21* |
| HZZ8-2_1 | *dfrA1-aadA1* | ST349 | IS*6-intI1-dfrA1-aadA1-**qacEΔ1-sul1-*IS*21* |
| HZZ8-3_1 | *dfrA1-aadA1* | ST349 | IS*6-intI1-dfrA1-aadA1-qacEΔ1-sul1-*IS*21* |
| HZZ8-4_1 | *dfrA1-aadA1* | ST349 | IS*6-intI1-dfrA1-aadA1-qacEΔ1-sul1-*IS*21* |
| HZZ8-5_1 | *dfrA1-aadA1* | ST349 | IS*6-intI1-dfrA1-aadA1-qacEΔ1-sul1-*IS*21* |
| HZZ7-1_1 | *dfrA1-aadA1* | ST349 | IS*6-intI1-dfrA1-aadA1-qacEΔ1-sul1-*IS*21* |
| HZDJ119_1 | *dfrA1-aadA1* | ST7285 | IS*6-intI1-dfrA1-aadA1-qacEΔ1-sul1-*IS*21* |
| LSSZ41 | *dfrA1-aadA1* | ST1431 | IS*1-intI1-dfrA1-aadA1-qacEΔ1-sul1**-*Tn*3* |
| SYF96_1 | *dfrA12-aadA2* | ST1196 | IS*6-intI1-dfrA12-aadA2- qacEΔ1-sul1-*IS*6* |
| SYF92_1 | *dfrA12-aadA2* | ST1196 | IS*6-intI1-dfrA12-aadA2- qacEΔ1-sul1-*IS*6* |
| SYF15_1 | *dfrA12-aadA2* | ST1196 | IS*6-intI1-dfrA12-aadA2- qacEΔ1-sul1-*IS*6* |
| SYF352_1 | *dfrA12-aadA2* | ST1196 | IS*6-intI1-dfrA12-aadA2- qacEΔ1-sul1-*IS*6* |
| HZZ5-13 | *dfrA12-aadA2* | ST201 | IS*6-intI1-dfrA12-aadA2-qacEΔ1-sul1-*IS*6* |
| LSSZ13 | *dfrA12-aadA2* | ST641 | IS*6-intI1-dfrA12-aadA2-qacEΔ1-sul1-*IS*91* |
| LSSZ64_1 | *dfrA12-aadA2* | ST641 | IS*6-intI1-dfrA12-aadA2-qacEΔ1-sul1-*IS*91* |
| LSSZ32_1 | *dfrA12-aadA2* | ST641 | IS*6-intI1-dfrA12-aadA2-qacEΔ1-sul1-*IS*91* |
| 85_1 | *dfrA12-aadA2* | ST3944 | IS*6-intI1-dfrA12-aadA2-qacEΔ1-sul1-*IS*6* |
| HZZ9-8_1 | *dfrA12-aadA2* | ST7508 | IS*6-intI1-dfrA12-aadA2-qacEΔ1-sul1-*IS*6* |
| 98_1 | *dfrA12-aadA2* | ST6422 | IS*6-intI1-dfrA12-aadA2-qacEΔ1-sul1-*IS*6* |
| HZZ7-2_1 | *dfrA12-aadA2* | ST2179 | IS*6-intI1-dfrA12-aadA2-qacEΔ1-sul1-*IS*6* |
| HZZ7-17_1 | *dfrA12-aadA2* | ST2179 | IS*6-intI1-dfrA12-aadA2-qacEΔ1-sul1-*IS*6* |
| QZZ21_1 | *dfrA12-aadA2* | ST165 | IS*6-intI1-dfrA12-aadA2-qacEΔ1-sul1-*IS*6* |
| HZRJ113_1 | *dfrA12-aadA2* | ST7508 | IS*6-intI1-dfrA12-aadA2-qacEΔ1-sul1-*IS*6* |
| QZDJ112_1 | *dfrA12-aadA2* | ST7508 | IS*6-intI1-dfrA12-aadA2-qacEΔ1-sul1-*IS*6* |
| HZZ89_1 | *dfrA7* | ST410 | IS*6-intI1- dfrA7-qacEΔ1-sul1-*IS*6* |
| HZZ3-3_1 | *dfrA7* | ST101 | IS*6-intI1- dfrA7-qacEΔ1-sul1-*IS*6* |
| HZZ29_1 | *dfrA7* | ST133 | Tn*3-intI1-dfrA7-qacEΔ1-sul1-*IS*21* |
| HZZ5-11_1 | *dfrA7* | ST133 | Tn*3-intI1-dfrA7-qacEΔ1-sul1-*IS*21* |
| QZDJ119_1 | *dfrA7* | ST101 | Tn*3-intI1-dfrA7-qacEΔ1-sul1-*IS*21* |
| LSSZ42_1 | *aac(6’)-Ib* | ST1914 | IS*6-intI1- aac(6’)-Ib-qacEΔ1-sul1-*IS*6* |
| QZDJ116_1 | *aac(6’)-Ib* | ST9022 | IS*6-intI1- aac(6’)-Ib-qacEΔ1-sul1-*IS*6* |
| HZDJ111_1 | *aac(6’)-Ib* | ST720 | IS*6-intI1- aac(6’)-Ib-qacEΔ1-sul1-*IS*6* |
| HZZ7-10_1 | *aadA1-aac(3)-VIa* | ST1286 | Tn*3-intI1-aadA1-aac(3)-VIa -*IS*91-*IS*256-qacEΔ1-sul1-*IS*110* |
| HZZ7-13_1 | *aadA1-aac(3)-VIa* | ST1485 | Tn*3-intI1-aadA1-aac(3)-VIa -*IS*91-*IS*256-qacEΔ1-sul1-*IS*110* |
