# Supplementary material for: Prevalence and characterization of class I integrons in multidrug-resistant Escherichia coli isolates from humans and food-producing animals in Zhejiang Province, China
Source: BMC Microbiol. 2025 Feb 15;25:76. doi: 10.1186/s12866-025-03794-y (PMC11830211; doi:10.1186/s12866-025-03794-y)
Supplement: Supplementary file 6 — Supplementary Material 6 [file 12866_2025_3794_MOESM6_ESM.docx]

**Table S3** The gene cassette arrays, sequence types and integron sequences of 34 non-classic class I integrons

| Sample ID | Gene cassette arrays | Sequence types | Integron sequences |
| --- | --- | --- | --- |
| HZZ84_1 | *aph(4)-Ia-aph(3’)-Ia* | ST457 | *intI1-aph(4)-Ia-aph(3’)-Ia-sul2* |
| HZZ85_1 | *aph(4)-Ia-aph(3’)-Ia* | ST457 | *intI1-aph(4)-Ia-aph(3’)-Ia-sul2* |
| F15_1 | *aph(3’)-Ia* | ST1121 | *intI1-aph(3’)-Ia-sul2* |
| HZDJ115_1 | *aph(4)-Ia-aph(3’)-Ia* | ST10 | *intI1-aph(4)-Ia-aph(3’)-Ia-sul2* |
| HZDJ113_1 | *aph(4)-Ia-aph(3’)-Ia* | ST10 | *intI1-aph(4)-Ia-aph(3’)-Ia-sul2* |
| QZDJ164_1 | *aph(4)-Ia-aph(3’)-Ia* | ST10 | *intI1-aph(4)-Ia-aph(3’)-Ia-sul2* |
| QZDJ139_1 | *aph(4)-Ia-aph(3’)-Ia* | ST10 | *intI1-aph(4)-Ia-aph(3’)-Ia-sul2* |
| QZRJ307_1 | *aph(4)-Ia-aph(3’)-Ia* | ST10 | *intI1-aph(4)-Ia-aph(3’)-Ia-sul2* |
| SYF302_1 | *aph(3’)-Ia* | ST744 | *intI1-aph(3’)-Ia-sul2* |
| SYF308_1 | *aph(3’)-Ia* | ST744 | *intI1-aph(3’)-Ia-sul2* |
| HZZ5-10_1 | *aph(3’)-Ia* | ST1196 | *intI1-aph(3’)-Ia-sul2* |
| HZZ83_1 | *aadA8* | ST88 | *intI1-aadA8-qacL-sul3* |
| LSSZ55_1 | *aadA1* | ST10 | *intI1-aadA1-qacL-sul3* |
| HZZ6-3_1 | *aadA1* | ST711 | *intI1-aadA1-qacL-sul3* |
| HZZ6-8_1 | *aadA1* | ST761 | *intI1-aadA1-qacL-sul3* |
| HZDJ112_1 | *aadA1* | ST281 | *intI1-aadA1-qacL-sul3* |
| HZDJ127_1 | *aadA1* | ST281 | *intI1-aadA1-qacL-sul3* |
| HZZ7-12_1 | *aac(3)-VIa-aph(4)-Ia* | ST443 | *intI1-aac(3)-VIa-aph(4)-Ia-sul2* |
| SYF322_1 | *dfrA1* | ST73 | *inI1-dfrA1* |
| HZZ6-11_1 | *dfrA1* | ST155 | *inI1-dfrA1* |
| HZZ6-12_1 | *dfrA5* | ST237114 | *inI1-dfrA5* |
| HZZ7-11_1 | *dfrA12* | ST237112 | *inI1-dfrA12* |
| HZZ7-14_1 | *dfrA12* | ST237112 | *inI1-dfrA12* |
| HZZ5-13_1 | / | ST641 | *intI1* |
| HZZ5-16_1 | / | ST133 | *intI1* |
| HZDJ114_1 | / | ST457 | *intI1* |
| HZDJ116_1 | / | ST744 | *intI1* |
| HZDJ117_1 | / | ST744 | *intI1* |
| LSSZ6-11_1 | / | ST237113 | *intI1* |
| HZZ6-18_1 | / | ST133 | *intI1* |
| QZDJ148_1 | / | ST165 | *intI1* |
| LSSZ13_1 | / | ST237113 | *intI1* |
| HZZ9-2_1 | / | ST237112 | *intI1* |
| HZZ9-10_1 | / | ST237112 | *intI1* |
